# Prevalence of Antimicrobial Resistance and Characteristics of *Escherichia coli* Isolates From Fecal and Manure Pit Samples on Dairy Farms in the Province of Québec, Canada

**DOI:** 10.3389/fvets.2021.654125

**Published:** 2021-05-21

**Authors:** Jonathan Massé, Hélène Lardé, John M. Fairbrother, Jean-Philippe Roy, David Francoz, Simon Dufour, Marie Archambault

**Affiliations:** ^1^Regroupement FRQNT Op+lait, Saint-Hyacinthe, QC, Canada; ^2^Groupe de Recherche sur les maladies infectieuses en production animale, Saint-Hyacinthe, QC, Canada; ^3^Department of Pathology and Microbiology, Faculty of Veterinary Medicine, Université de Montréal, Saint-Hyacinthe, QC, Canada; ^4^Department of Clinical Sciences, Faculty of Veterinary Medicine, Université de Montréal, Saint-Hyacinthe, QC, Canada

**Keywords:** antimicrobial resistance, *Escherichia coli*, ESBL/AmpC, fecal, dairy cattle, calf, manure pit

## Abstract

Antimicrobial resistance (AMR) is an important burden for public health and veterinary medicine. For Québec (Canada) dairy farms, the prevalence of AMR is mostly described using passive surveillance, which may be misleading. In addition, the presence of extended spectrum β-lactamase (ESBL)/AmpC producing *Escherichia coli* is unknown. This observational cross-sectional study used random dairy farms (*n* = 101) to investigate AMR and extended spectrum β-lactamase (ESBL)/AmpC producing *Escherichia coli*. Twenty antimicrobials were tested on *E. coli* isolates (*n* = 593) recovered from fecal samples (*n* = 599) from calves, cows, and the manure pit. Isolates were mostly susceptible (3% AMR or less) to the highest priority critically important antimicrobials in humans. The highest levels of AMR were to tetracycline (26%), sulfisozaxole (23%) and streptomycin (19%). The resistance genes responsible for these resistances were, respectively*: tet*(A)*, tet*(B), *sul*1*, sul*2*, sul*3*, aph(*3”*)-Ib (str*A*), aph(*6*)-Id (str*B*), aad*A1*, aad*A2, *and aad*A5. ESBL analysis revealed two predominant phenotypes: AmpC (51%) and ESBL (46%) where *bla*_CMY−2_ and *bla*_CTX−M__(_*bla*_CTX−M−1_, *bla*_CTX−M−15_, and *bla*_CTX−M−55)_ were the genes responsible for these phenotypes, respectively. During this study, 85% of farms had at least one ESBL/AmpC producing *E. coli*. Isolates from calves were more frequently resistant than those from cows or manure pits. Although prevalence of AMR was low for critically important antimicrobials, there was a high prevalence of ESBL/AmpC-producing *E. coli* on Quebec dairy farms, particularly in calves. Those data will help determine a baseline for AMR to evaluate impact of initiatives aimed at reducing AMR.

## Introduction

Antimicrobial resistance (AMR) is an important public health concern (1, 2). Indeed, around the world, AMR has major financial and health implications for humans, animals and the environment. According to an OIE report, this burden will dramatically increase the number of human deaths in the future (3). A multitude of national programs now ensures surveillance of AMR data for several bacterial species in humans and animals and publishes their reports annually [e.g., Canada (CIPARS), USA (NARMS), Denmark (DANMAP)]. However, some surveillance programs are incomplete as data originated from slaughterhouses or diagnostic laboratories and may not accurately reflect the real on farm conditions. The present AMR situation in dairy farms is currently unknown in Canada, including in the province of Québec.

To investigate AMR in healthy animals, commensal bacteria, such as *Escherichia coli*, is globally used as an indicator to estimate AMR in livestock (4). In addition, in dairy animals, the age of the animal has been reported as an important determinant of AMR carriage. Calves can carry higher levels of AMR in fecal *E. coli* isolates compared to older animals (5). Furthermore, feces from animals is an important hazard for spread of resistant organisms in the environment (6) and eventually to humans, as manure is used to fertilize crops. The composition of the population of *E. coli* isolates found in feces may be affected by the animal's living conditions such as geographic region or purpose (dairy or beef) (7, 8) and by the antimicrobial treatments it has received (8, 9). However, when manure is commingled in the manure pit, the AMR profiles of the indicator *E. coli* may be further influenced by environmental conditions such as multiple freeze-thaw cycles (10). Consequently, it would be useful to test the manure pit to investigate this possibility.

Extended-spectrum β-lactamase (ESBL)/AmpC-producing *Enterobacteriacea* are a growing public health concern in veterinary medicine (11). The World Health Organization has recently published a global priority pathogens list to focus attention on the most significantly resistant pathogens. ESBL-producing *Enterobacteriaceae* are included within the critical category of this list (12). ESBL/AmpC *Enterobacteriaceae* are resistant to penicillin, second- and third-generation cephalosporins, and monobactams (13). ESBL are associated with resistance to fourth-generation cephalosporins, although β-lactamase inhibitors usually neutralize their activity. AmpC are associated with susceptibility to fourth-generation cephalosporins, but with resistance to cephamycins and β-lactamase inhibitors (13). Despite their public health importance, fecal carriage of ESBL/AmpC is not well-described in dairy animals in Canada.

The objective of this paper was, therefore, to investigate the prevalence of AMR and of ESBL/AmpC in *E. coli* isolated from fecal samples in calves, cows, and from the manure pits on Québec dairy farms.

## Materials and Methods

### Selection of Herds and Sample Collection

The current study was an observational cross-sectional study on commercial dairy farms. The research protocol was approved prior to initiating the research by the Animal Use Ethics Committee of the Université de Montréal (Protocol 16-Rech-1859). The complete protocol describing sample size calculation, exclusion criteria, recruitment of participants, and method used to avoid selection bias is available elsewhere (14). Briefly, dairy farms from a random list of dairies in three regions of Québec province were solicited for participation by telephone between January and March 2017. Participation was on a voluntary basis. Basic demographic information was obtained from producers refusing to participate to quantify any selection bias. Given that some producers may decide to leave the study during the study period, we aimed at recruiting 102 farms (vs. our sample size estimate of 100 farms). We use a stratified random sampling to achieve a proportion of herds similar to the source population: 45 (45%) herds in the Montérégie region, 35 (34%) herds in the Centre-du-Québec region and 22 (21%) herds in the Estrie region.

Following recruitment, two sampling visits were made, firstly between April and June 2017 and secondly between October and November 2017. On each visit, three composite fecal samples were collected from: five randomly chosen pre-weaned calves; five randomly chosen lactating cows; and two convenient locations in the manure pit. The total number of calves and lactating cows was recorded upon arrival and a random number generator was used to select five animals in each group for sample collection. If fewer than five pre-weaned calves were present on the farm, all available pre-weaned calves were sampled. Fecal samples were collected directly from the rectum of each individual animal. If the manure pit was inaccessible, the last indoor point before the manure pit or the conveyer was used. For each of these three composite samples, approximately 25 g of composite feces or manure was placed in a 50 mL sterile tube and stored immediately on ice at the farm. Samples were processed in the laboratory within <24 h. A preservative medium (peptone water with 30% glycerol) was added to feces at a 1:1 volume-to-weight ratio; samples were then homogenized and frozen at −70°C.

### Bacterial Isolation

One gram of thawed composite fecal samples was mixed in 9 mL of phosphate buffer saline. A volume as determined by previous standardization in our laboratory, (1uL for calves, 10uL for cows and 100uL for manure pits) was spread on MacConkey agar (Oxoid, Canada) then incubated overnight at 37°C. One lactose positive colony was subcultured on Columbia agar with 5% sheep blood (Oxoid, Canada), and then incubated overnight at 37°C. The identification of isolates as *E. coli* was confirmed by MALDI-TOF MS using a Microflex LT instrument and the reference spectra database from Brucker containing 7,311 spectra (Bruker Daltonics, Germany).

### Antimicrobial Susceptibility Testing

The minimum inhibitory concentrations (MIC) for 20 antimicrobials (Table 1) representing 11 classes of antimicrobials were determined with the broth microdilution method using commercially available panels (Sensititre CMV4AGNF and BOPO6F) (Thermo Fisher scientific, Canada) following manufacturer recommendations in accordance with CLSI. Isolates were defined as susceptible, intermediate, or resistant according to CLSI M100 (16) (*Enterobacteriaceae*: amoxicillin/clavulanate, azithromycin, ampicillin, cefoxitin, ceftriaxone, chloramphenicol, ciprofloxacin, gentamicin, meropenem, nalidixic acid, sulfisoxazole, tetracycline and trimethoprim/sulfamethoxazole), CLSI VET08 (17) (ceftiofur, danofloxacin, enrofloxacin and spectinomycin), or CIPARS (18) (streptomycin) clinical breakpoints. A MIC breakpoint was not available for neomycin, thus the epidemiological cut-off value from European Committee on Antimicrobial Susceptibility Testing (EUCAST) was used (MIC ≥ 16 μg mL^−1^ was defined as resistant). There was no valid florfenicol clinical breakpoint for *Enterobacteriaceae* and the tested concentrations (0.25–4 μg mL^−1^) did not include the European epidemiological cut-off of 16 μg mL^−1^, therefore no interpretation was attempted. For subsequent analyses, intermediate and resistant isolates were grouped together and labeled as resistant. Multidrug resistance (MDR) was defined as acquired resistance to at least one agent in three or more antimicrobial classes (19). *Enterococcus faecalis* ATCC 29212, *Escherichia coli* ATCC 25922, *Staphylococcus aureus* ATCC 29213 and *Pseudomonas aeruginosa* ATCC 27853 were used as reference strains for batch controls. *Escherichia coli* ATCC 25922 was used as a daily control.

**Table 1:** Minimum inhibitory concentration for medically important antimicrobials, according to the WHO, of 593 *Escherichia coli* isolated from calf or cow feces or manure pit of 101 dairy farms in Québec Canada.

**Importance for human medicine^**†**^**	**Antimicrobial Class**	**Antimicrobial Agent**	**MIC (μg ml**^****−1****^)^*^																**%** **resistant**
			**0.015**	**0.03**	**0.06**	**0.12**	**0.25**	**0.5**	**1**	**2**	**4**	**8**	**16**	**32**	**64**	**128**	**256**	**512**	
Critically important	Cephalosporin	Ceftiofur					38.4	57.8	0.5	0.8	-8,-515pt0.7	0.5	1.2						2.4
– Highest priority	3rd generation	Ceftriaxone					96.3	0.5	0.2	-8,-515pt0.7		1.0	0.5			0.8			3.0
	Quinolone	Ciprofloxacin	97.3	1.3	0.3	0.2	0.8			-8,-515pt									0
		Danofloxacin				99.0	0.3	-8,-515pt0.5	0.2										0.7
		Enrofloxacin				99.0	0.5	-8,-515pt 0.3	0.2										0.5
		Nalidixic acid							5.4	74.4	19.1	0.3	0.2		0.7				0.7
	Macrolide	Azithromycin						0.2	1.2	19.6	68.6	7.6	0.5	1.3	1.0				2.3
Critically important	Aminoglycoside	Gentamicin					2.0	70.0	25.8	0.7		-8,-515pt	0.2	1.3					1.5
– High priority		Neomycin									91.1	0.5	0.2	1.3	6.9				8.4
		Streptomycin								0.3	43.0	33.4	1.3	2.5	5.7	13.7			19.4
	Carbapenem	Meropenem			99.7	0.3				-8,-515pt									0
	Amino/β-lac inh^‡^	Amox.-CLA^‡^							2.4	17.4	63.1	14.2	-8,-515pt 0.5	2.0	0.5				3.0
	Aminopenicillin	Ampicillin							5.6	42.7	36.3	1.7	-8,-515pt		13.8				13.8
Highly important	Cephamycin	Cefoxitin							0.2	6.2	68.6	21.8	-8,-515pt1.0	0.8	1.3				3.2
	Folate pathway	Sulfisoxazole											43.5	31.5	2.4			22.6	22.6
	antagonist	TMP-sulfa^§^				81.5	5.4	0.7		0.2		12.3							12.3
	Phenicol	Chloramphenicol								0.8	26.0	62.6	-8,-515pt 0.7		9.9				10.6
		Florfenicol^¶^							0.2	5.9	68.5	16.7	8.8						NA
	Tetracycline	Tetracycline									74.4	-8,-515pt 0.7	0.5	1.9	22.6				25.6
Important	Aminocyclitol	Spectinomycin										4.4	75.9	9.4	-8,-515pt 2.0	8.3			10.3

* *Numbers indicate percentages of isolates. White areas are concentrations of antimicrobials tested by the broth microdilution method. Percentages in gray areas have a MIC superior to the concentration range tested. Percentages in the first white area starting from left have MIC inferior or equal to the corresponding concentration. Dashed and plain lines represent threshold used to define intermediate and resistant clinical breakpoints, respectively. Intermediate and resistant isolates were grouped together and labeled as resistant for the last column of the table*;

† *Importance of antimicrobials according to World Health Organization (15)*.

‡ *Penicillin + β-lactamase inhibitor: Amoxicillin/clavulanic acid combination*;

§ *Trimethoprim-sulfamethoxazole combination*;

¶ *Florfenicol has no valid clinical breakpoints for Enterobacteriaceae and the concentration of 0.25 to 4μg mL^−1^ did not include the European epidemiological breakpoint of 16μg mL^−1^, thus no interpretation could be given*.

### Isolation of Presumptive ESBL/AmpC-Producing *E. coli* and Phenotypic Confirmation

**Figure 1:**
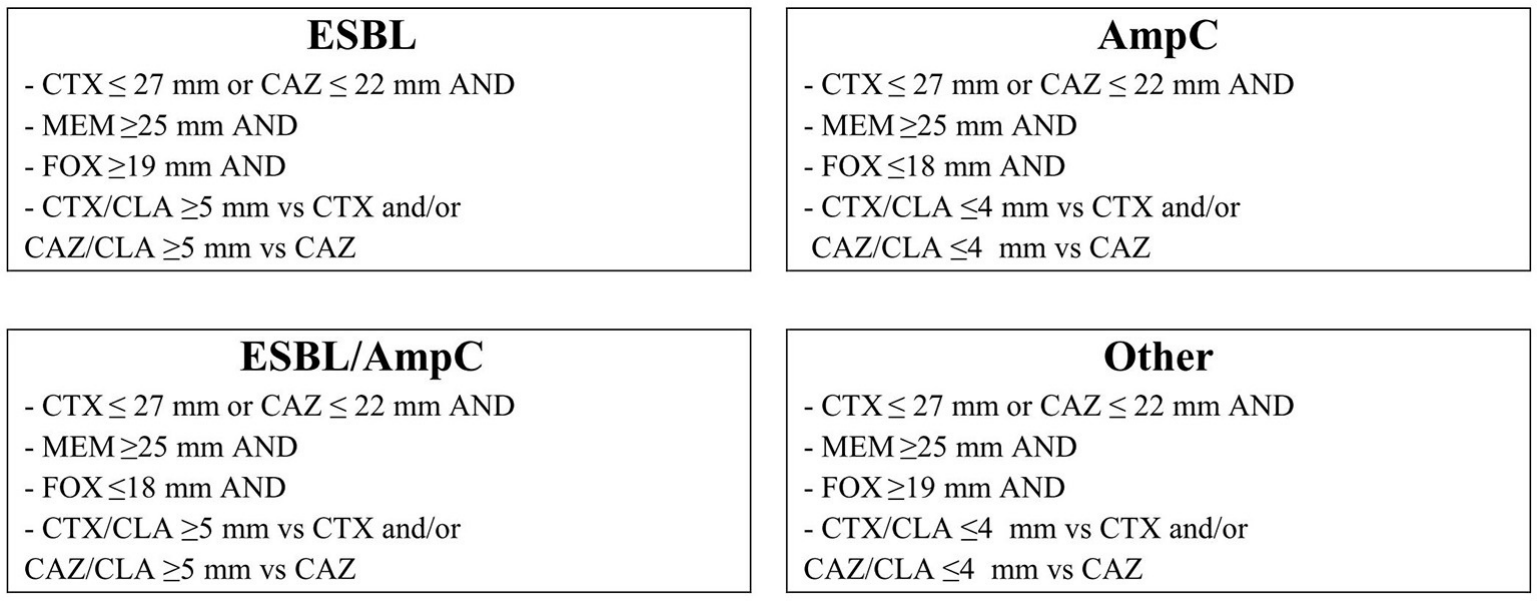
ESBL/AmpC phenotypes by disk diffusion method derived from MIC by the European Committee on Antimicrobial Susceptibility Testing definition. CTX, CAZ, CTX/CLA, and CAZ/CLA are zone diameters from CLSI VET08 table 7A. FOX and MEM are zone diameters from EUCAST guidelines for detection of resistance mechanisms and specific resistances of clinical and/or epidemiological importance. CAZ ceftazidime (30 μg); CLA clavulanate (10 μg); CTX cefotaxime (30 μg); FOX cefoxitin (30 μg); MEM meropenem (10 μg).

Fecal samples were processed according to the laboratory protocol of the European Union Reference Laboratory on Antimicrobial Resistance which allow the isolation of ESBL-, AmpC- and carbapenemase-producing *E. coli* from fecal samples after a discussion with this laboratory. The protocol is available online at https://www.eurl-ar.eu/protocols.aspx. Briefly, 1 g of feces or manure was added to 9 mL of Buffered Peptone Water, then incubated at 37 °C for 20 h. One loop (10 μl) was streaked onto a MacConkey agar plate containing 1 mg mL^−1^ of cefotaxim, then incubated at 44 °C for 20 h. Lactose positive colonies were subcultured on Columbia agar with 5% sheep blood, and then incubated overnight at 37°C. Identification of *E. coli* was confirmed by MALDI-TOF MS. Samples with at least one *E. coli* colony isolated with this technique were labeled as presumptive ESBL/AmpC *E. coli*. Isolate 2005-60-10-96-1 (MIC cefotaxim: 2 μg mL^−1^) and isolate OXA-30 (MIC cefotaxim: 0.5 μg mL^−1^) were used as positive and negative controls, respectively. Secondly, ESBL production was confirmed according to the Clinical Laboratory Standard Institute (CLSI) protocol disk diffusion tests for ESBL in *E. coli* because this technique is the gold standard assay of the CLSI (17). In addition, cefoxitin and meropenem disks were added to investigate AmpC ß-lactamase and meropenemase production. The phenotype assignments (definitions shown in Figure 1) were defined according to EUCAST (20) and CLSI cut-offs (17). *Klebsiella pneumoniae* ATCC 700603 and *E. coli* ATCC 25922 were used as positive and negative controls, respectively.

### Antimicrobial Genotyping

Whole genome sequencing (WGS) was used on a subset of isolates (*n* = 16) to determine the genetic basis of the observed AMR. Isolates resistant to nine or more antimicrobials classes (aminoglycosides and aminocyclitols were considered two different classes for this selection) (*n* = 8) were selected. Furthermore, isolates identified as harboring an ESBL (*n* = 4) or an AmpC (*n* = 4) phenotype were randomly selected. Briefly, genomic DNA was extracted using QIAamp DNA Mini Kit for DNA following manufacturer's guidelines (Qiagen, Hilden, Germany). We performed WGS on the Illumina (San Diego, CA) MiSeq platform with 2 × 300 paired-end runs after library preparation with the Illumina Nex-tera XT DNA Library preparation kit, according to the manufacturer's instructions. Galaxy (https://usegalaxy.org/) (21) platform was used for *in-silico* analysis, assembled genomes were obtained using SPADES software (Galaxy Version 3.12.0+galaxy1) (22) and assembly quality was evaluated with Quast (Galaxy Version 5.0.2+galaxy1) (23). An assembly was rejected if the number of contigs was > 410, if the N50 was <40,000 or if the number of contigs were between 300 and 400 and the N50 <50,000. To search AMR genes and point mutations, Res Finder 4.0 (24) and Point Finder (25) bioinformatics tools were used from the Center of Genomic Epidemiology (CGE) platform (http://www.genomicepidemiology.org/).

### Statistical Analyses

For all statistical analyses, the unit of analysis was the sample obtained from a given source (calves, cows, or manure pit), time (autumn or spring visit), and herd. These samples were represented by one *E. coli* isolate.

#### Effect of Sample Origin on Prevalence of Resistant Isolates

We investigated whether probability of resistance to a given antimicrobial differed between isolates obtained from pre-weaned calves, cows, or the manure pit. To achieve this, results from up to two *E. coli* isolates (one of the autumn and one of the spring visit) were available for each sample type (calves, cows, manure pits) and for each herd. For this analysis, we used a logistic regression model with susceptibility *vs*. resistance to a given antimicrobial as outcome variable, sample type as sole fixed predictor, and estimated using robust variance to account for clustering of isolates by herd (SAS, PROC GENMOD. Cary, NC, US). Whenever this model could not converge, a Fisher exact test was used, thus ignoring clustering of observations. Tukey-kramer adjustment was used to adjust for multiple comparisons. An alpha of 0.05 was chosen to define statistically significant results.

#### Effect of Sample Origin and Season on Number of Antimicrobials to Which an Isolate Was Resistant

A generalized linear mixed model (SAS, PROC GLIMMIX, Cary, NC, US) was used to investigate whether the origin of the samples (calves, cows, or manure pit) or the season (autumn or spring) could influence the number of antimicrobials to which an isolate was resistant. In this model, a Poisson distribution with a log link was used. The outcome was the number of antimicrobial classes to which an isolate was defined as resistant (0, 1, 2, 3, …). The predictor was either the origin of the samples (calves, cows, or manure pits) or the season (autumn or spring) and a random herd intercept was included to account for clustering of isolates by herd. A Tukey-Kramer test was applied to adjust for multiple comparisons and an alpha of 0.05 was used.

#### Recovery of Presumptive ESBL/AmpC-Producing *E. coli*

A generalized mixed model with a logit link was used to investigate probability of recovery of an ESBL/AmpC isolate. In the first model, the outcome was recovery or not of an ESBL/AmpC isolate in a given sample, and we accounted for clustering of observations by visit and herd by including random visit and random herd intercepts, respectively. In this first model, however, we did not include any fixed predictors (i.e., a null model). The variance estimates obtained from this model were used to partition the outcome's variance using the simulation method described previously (26). This allowed for reporting the proportion of the risk of observing an ESBL/AmpC isolate that was due to sample', visit', or herd's characteristics. This model was then used to estimate the effect of sample source and of season on probability of recovery of an ESBL/AmpC isolate, simply by including these predictors in the model one at a time.

Finally, we investigated whether the recovery of an ESBL/AmpC isolate was associated with the number of antimicrobial resistances observed in this isolate. The Poisson generalized mixed model described in the preceding section was used with recovery of an ESBL/AmpC isolate (yes or no) as sole fixed predictor and number of resistances as outcome. A Tukey-Kramer test was applied for all analyses to adjust for multiple comparisons and an alpha of 0.05 was used.

## Results

### Sample Collection

We recruited 102 dairy farms. Only one farm left the study early after the first sampling visit and was excluded from our analyses. Four and three farms had no pre-weaned calves present on site at the first and second visits, respectively. On the first visit, we sampled 325 pre-weaned calves (average: 29 days old, range: 1 to 150), and 505 lactating cows (average: 2.6 lactation, range: 1 to 9). On the second visit, we sampled 395 pre-weaned calves (average: 27 days old, range: 1 to 100) and 505 lactating cows (average: 2.7 lactation, range: 1–10). Manure pits were emptied approximately 7 and 3 months before the first and second visits, respectively. In Québec province, manure pits are generally emptied at the beginning of autumn (October) and spring (May). Hence, the majority of manure pits were full on the first visit (spring) and nearly empty on the second visit (autumn). Among the 599 fecal composite samples obtained, we recovered 593 *E. coli* isolates as six samples from manure pits did not yield any lactose positive colonies.

### Antimicrobial Resistance Prevalence, Phenotypes and Predominant AMR Genes

**Figure 2:**
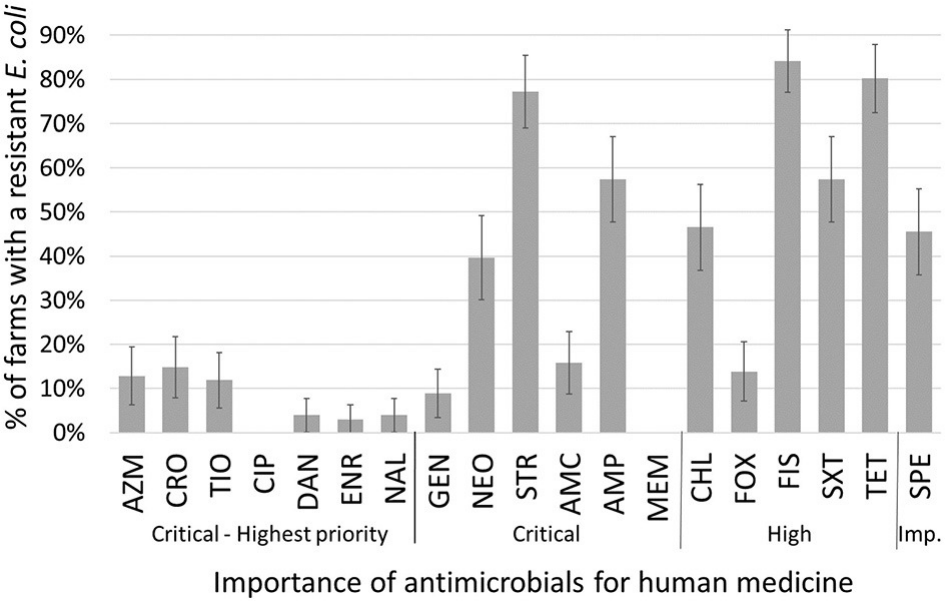
Proportion of farms with a least one resistant (intermediate and resistant combined) *Escherichia coli* from any sample or any season from 101 dairy farms from Québec, Canada. On each farm, between 4 and 6 *E. coli* were tested for each antimicrobial. Importance of antimicrobial for human medicine according to World Health Organization (15). AMC amoxicillin-clavulanic acid; AMP, ampicillin; AZM, azithromycin; CHL, chloramphenicol; CIP, ciprofloxacin; CRO, ceftriaxone; DAN, danofloxacin; ENR, enrofloxacin; FIS, sulfisoxazole; FOX, cefoxitin; GEN, gentamicin; MEM, meropenem; NAL, nalidixic acid; NEO, neomycin; SPT, spectinomycin; STR, streptomycin; SXT, trimethoprim-sulfamethoxazole; TET, tetracycline; TIO, ceftiofur.

Most isolates (70%; 414/593) were susceptible to all antimicrobials tested (Table 1). All isolates were susceptible to meropenem and ciprofloxacin, which are of critical importance in human medicine (15). A low level of AMR ( ≤ 3%) was observed to highest priority critically important antimicrobials (ceftriaxone, ceftiofur, danofloxacin, enrofloxacin, ciprofloxacin, nalidixic acid, and azithromycin). The highest levels of resistance were to tetracycline (26%), sulfisoxazole (23%), and streptomycin (19%). These highest levels of resistance were also observed at the farm level (Figure 2). The most prevalent resistance patterns were: tetracycline alone (4%), tetracycline-streptomycin-sulfisoxazole (3%), and chloramphenicol-tetracycline-streptomycin-sulfisoxazole (2%) (Table 2). Twenty-two isolates were resistant to six or more antimicrobial classes and were observed in 19 different patterns (Table 2). The resistance genes responsible for the highest levels of AMR toward tetracycline, sulfisozaxole, and streptomycin were, respectively*: tet*(A)*, tet*(B), *sul*1*, sul*2*, sul*3*, aph(*3”*)-Ib (str*A*), aph(*6*)-Id (str*B*), aad*A1*, aad*A2, *and aad*A5 (Table 3).

**Table 2:** Antimicrobial resistance patterns of 593 *Escherichia coli* isolated from calf or cow feces or manure pit of 101 dairy farms in Québec Canada.

**Antimicrobial pattern**	**Number of isolates (%)**
Pan-Susceptible	414 (69.8)
TET	24 (4.0)
SPT	4 (0.7)
FOX	3 (0.5)
CHL	2 (0.3)
STR	1 (0.2)
FIS	1 (0.2)
AMP	1 (0.2)
FIS, SXT	1 (0.2)
GEN, SPT	1 (0.2)
FIS, TET	4 (0.7)
AMP, TET	2 (0.3)
AMP, STR	1 (0.2)
FOX, CHL	1 (0.2)
AZM, FIS	1 (0.2)
AMP, FIS	1 (0.2)
AMP, TIO, CRO	1 (0.2)
STR, FIS, TET ^MDR^	16 (2.7)
NEO, FIS, TET ^MDR^	1 (0.2)
AMP, SPT, TET ^MDR^	1 (0.2)
AMP, GEN, TET ^MDR^	1 (0.2)
AMP, CHL, FIS ^MDR^	1 (0.2)
AMP, FIS, TET ^MDR^	1 (0.2)
AMC, AMP, FOX ^MDR^	1 (0.2)
SPT, STR, FIS, TET ^MDR^	3 (0.5)
SPT, FIS, TET, SXT ^MDR^	2 (0.3)
NEO, STR, FIS, TET ^MDR^	2 (0.3)
AMP, STR, FIS, SXT ^MDR^	2 (0.3)
AMP, SPT, FIS, SXT ^MDR^	1 (0.2)
AMP, NEO, STR, FIS ^MDR^	1 (0.2)
AMP, TIO, CRO, TET ^MDR^	1 (0.2)
CHL, STR, FIS, TET ^MDR^	11 (1.9)
AMP, CHL, FIS, TET ^MDR^	1 (0.2)
NEO, STR, FIS, TET, SXT ^MDR^	1 (0.2)
AMP, GEN, STR, FIS, SXT ^MDR^	1 (0.2)
AMP, STR, FIS, TET, SXT ^MDR^	6 (1.0)
CHL, NEO, STR, FIS, TET ^MDR^	3 (0.5)
AMP, SPT, STR, FIS, TET ^MDR^	2 (0.3)
AMP, NEO, STR, FIS, TET ^MDR^	2 (0.3)
AMP, SPT, FIS, TET, SXT ^MDR^	1 (0.2)
CHL, STR, FIS, TET, SXT ^MDR^	1 (0.2)
AMP, NEO, SPT, FIS, TET ^MDR^	1 (0.2)
AMP, CHL, STR, FIS, TET ^MDR^	3 (0.5)
AMP, CHL, SPT, FIS, TET ^MDR^	1 (0.2)
NEO, SPT, STR, FIS, TET, SXT ^MDR^	4 (0.7)
CHL, SPT, STR, FIS, TET, SXT ^MDR^	4 (0.7)
AMP, NEO, STR, FIS, TET, SXT ^MDR^	3 (0.5)
AMP, SPT, STR, FIS, TET, SXT ^MDR^	1 (0.2)
AMP, CHL, STR, FIS, TET, SXT ^MDR^	1 (0.2)
AMP, NEO, SPT, STR, FIS, TET, SXT ^MDR^	2 (0.3)
AMP, TIO, CRO, SPT, STR, FIS, SXT ^MDR^	1 (0.2)
AMP, CHL, SPT, STR, FIS, TET, SXT ^MDR^	4 (0.7)
AMP, CHL, NEO, STR, FIS, TET, SXT ^MDR^	4 (0.7)
AMP, AZM, SPT, STR, FIS, TET, SXT ^MDR^	2 (0.3)
AMC, AMP, GEN, NEO, STR, FIS, TET ^MDR^	1 (0.2)
CHL, DAN, NAL, SPT, STR, FIS, TET ^MDR^	1 (0.2)
AMP, NAL, NEO, STR, FIS, TET, SXT ^MDR^	1 (0.2)
AZM, CHL, SPT, STR, FIS, TET, SXT ^MDR^	1 (0.2)
AZM, CHL, NEO, STR, FIS, TET, SXT ^MDR^	1 (0.2)
AMP, CHL, GEN, SPT, STR, FIS, TET ^MDR^	1 (0.2)
AMC, AMP, CHL, NEO, SPT, FIS, TET ^MDR*^	1 (0.2)
DAN, ENR, NEO, SPT, STR, FIS, TET, SXT ^MDR^	1 (0.2)
AMP, CHL, NEO, SPT, STR, FIS, TET, SXT ^MDR^	4 (0.7)
AZM, CHL, NEO, SPT, STR, FIS, TET, SXT ^MDR^	2 (0.3)
AMP, AZM, NEO, SPT, STR, FIS, TET, SXT ^MDR^	1 (0.2)
AMP, AZM, CHL, NEO, STR, FIS, TET, SXT ^MDR*^	2 (0.3)
AMP, AZM, CHL, NEO, SPT, FIS, TET, SXT ^MDR*^	1 (0.2)
AMC, AMP, FOX, GEN, NEO, SPT, FIS, TET, SXT ^MDR*^	1 (0.2)
AMP, AZM, TIO, CRO, SPT, STR, FIS, TET, SXT ^MDR*^	1 (0.2)
AMC, AMP, FOX, TIO, CRO, NEO, STR, FIS, TET ^MDR*^	1 (0.2)
AMC, AMP, FOX, TIO, CRO, SPT, STR, FIS, TET, SXT ^MDR*^	1 (0.2)
AMC, AMP, FOX, TIO, CRO, NEO, STR, FIS, TET, SXT ^MDR*^	1 (0.2)
AMC, AMP, FOX, CRO, CHL, SPT, STR, FIS, TET, SXT ^MDR*^	2 (0.3)
AMC, AMP, FOX, CRO, CHL, NEO, STR, FIS, TET, SXT ^MDR*^	1 (0.2)
AMC, AMP, FOX, TIO, CRO, CHL, STR, FIS, TET, SXT ^MDR*^	1 (0.2)
AMP, AZM, TIO, CRO, GEN, NEO, SPT, STR, FIS, TET, SXT ^MDR*^	1 (0.2)
AMC, AMP, CHL, DAN, ENR, NAL, SPT, STR, FIS, TET, SXT ^MDR*^	1 (0.2)
AMP, FOX, TIO, CRO, CHL, NEO, SPT, STR, FIS, TET, SXT ^MDR*^	1 (0.2)
AMC, AMP, FOX, TIO, CRO, CHL, SPT, STR, FIS, TET, SXT ^MDR*^	1 (0.2)
AMC, AMP, FOX, CRO, CHL, NEO, SPT, STR, FIS, TET, SXT ^MDR*^	1 (0.2)
AMC, AMP, FOX, TIO, CRO, CHL, NEO, STR, FIS, TET, SXT ^MDR*^	1 (0.2)
AMC, AMP, FOX, TIO, CRO, CHL, NEO, SPT, STR, FIS, TET, SXT ^MDR*^	1 (0.2)
AMC, AMP, CHL, DAN, ENR, GEN, NAL, NEO, SPT, STR, FIS, TET, SXT ^MDR*^	1 (0.2)
AMC, AMP, AZM, FOX, TIO, CRO, CHL, GEN, NEO, SPT, STR, FIS, TET, SXT ^MDR*^	1 (0.2)

MDR *Multidrug resistance was defined by resistance to three or more antimicrobial classes*.

* *Pattern resistant to six or more antimicrobial classes*.

*AMC, amoxicillin-clavulanic acid; AMP, ampicillin; AZM, azithromycin; CHL, chloramphenicol; CRO, ceftriaxone; DAN, danofloxacin; ENR, enrofloxacin; FIS, sulfisoxazole; FOX, cefoxitin; GEN, gentamicin; NAL, nalidixic acid; NEO, neomycin; SPT, spectinomycin; STR, streptomycin; SXT, trimethoprim-sulfamethoxazole; TET, tetracycline; TIO, ceftiofur*.

**Table 3:** Phenotypic resistance and presence of associated antimicrobial resistance genes for the most multidrug resistant *Escherichia coli* (*n* = 8) isolated from calf of 101 dairy farms in Québec, Canada.

**Antimicrobial**		**Isolate**							
**Class**	**Agent**	**10040013**	**10420013**	**10500013**	**10600013**	**10780027**	**10830027**	**10940027**	**10990027**
Aminocyclitol	SPT	*aad*A1	*aad*A1, *aad*A2	*aad*A1, *aad*A2	*aad*A1	*aad*A2	*aad*A2	*aad*A1	*aad*A5
Aminoglycoside	GEN					*aac(3)-IId*			*aac(3)-IId*
	NEO				*aph(3')-Ia*	*aph(3')-Ia*	*aph(3')-Ia*		*aph(3')-Ia*
	STR	*aad*A1 *strA/strB*	*aad*A1, *aad*A2	*aad*A1, *aad*A2 *strA/strB*	*aad*A1 *strA/strB*	*aad*A2 *strA/strB*	*aad*A2 *strA/strB*	*aad*A1 *strA/strB*	*aad*A5 *strA/strB*
Aminopenicillin	AMP	*bla_*CMY*−2_, bla_*TEM*−1*B*_*	*bla_*CARB*−2_*	ampC^*^	*bla_*CMY*−2_ bla_*TEM*−1*B*_*	*bla_*CMY*−2_ bla_*TEM*−1*B*_*	ampC^*^	ampC^*^	*bla_*TEM*−1*B*_*
Amino/β-lac inh^‡^	AMC	*bla_*CMY*−2_*		ampC^*^	*bla_*CMY*−2_*	*bla_*CMY*−2_*	ampC^*^	ampC^*^	
Cephamycin	FOX	*bla_*CMY*−2_*		ampC^*^	*bla_*CMY*−2_*	*bla_*CMY*−2_*	ampC^*^	ampC^*^	
Cephalosporin	TIO	*bla_*CMY*−2_*		ampC^*^	*bla_*CMY*−2_*	*bla_*CMY*−2_*	ampC^*^	ampC^*^	
	CRO	*bla_*CMY*−2_*		ampC^*^	*bla_*CMY*−2_*	*bla_*CMY*−2_*	ampC^*^	ampC^*^	
Folate pathway	FIS	*sul*2	*sul3*	*sul1, sul*2	*sul*2	*sul, sul*2	*sul, sul*2	*sul*2	*sul, sul*2
antagonist	SXT	*dfr*A1	*dfr*A16	*dfr*A23	*dfr*A1	*dfr*A12	*dfr*A23	*dfr*A1	*dfr*A17
Macrolide	AZM					*mph*(A)			
Phenicol	CHL	*floR*	*cml*A1^†^	*floR*	*floR*	*floR*	*floR*	*floR*	*floR*
Quinolone	DAN		*gyrA*^†^						*gyrA*^†^
	ENR		*gyrA*^†^						*gyrA*^†^
	NAL		*gyrA*^†^						*gyrA*^†^
Tetracycline	TET	*tet*(B)	*tet*(A)	*tet*(A)	*tet*(B)	*tet*(A), *tet*(B)	*tet*(A), *tet*(B)	*tet*(A)	*tet*(A)

*Phenotypic resistance is represented by dark and light gray cells for intermediate and resistant isolates, respectively. White cells are for susceptible isolates. Resistance genes indicated in cells denote their presence for a particular isolate*.

* *Promoter mutation in ampC−42 C -> T*;

† *Mutation in gyrA S83L*;

‡ *Penicillin +β-lactamase inhibitor*.

*AMC, amoxicillin-clavulanic acid; AMP, ampicillin; AZM, azithromycin; CHL, chloramphenicol; CRO, ceftriaxone; DAN, danofloxacin; ENR, enrofloxacin; FIS, sulfisoxazole; FOX, cefoxitin; GEN, gentamicin; NAL, nalidixic acid; NEO, neomycin; SPT, spectinomycin; STR, streptomycin; SXT, trimethoprim-sulfamethoxazole; TET, tetracycline; TIO, ceftiofur*.

### Prevalence Distribution of Resistant Isolates

**Figure 3:**
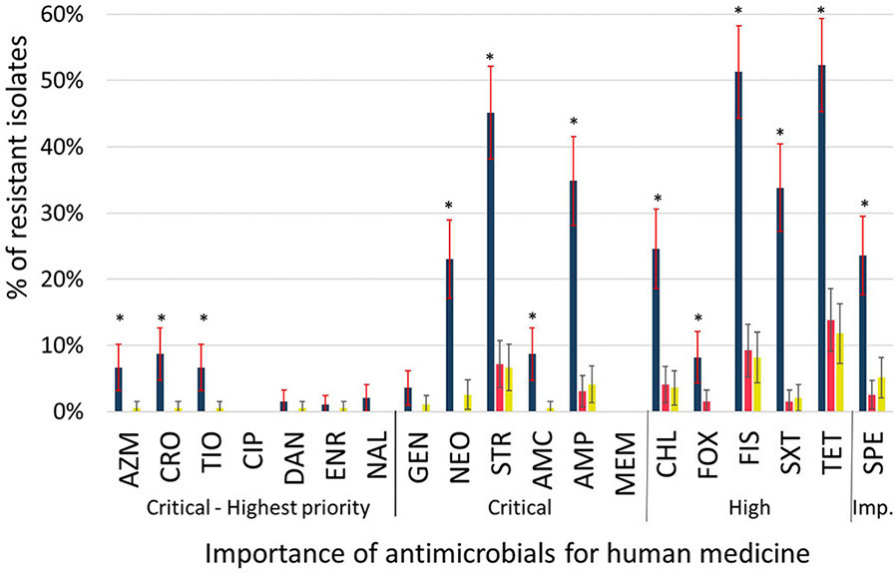
Proportion of resistant (intermediate and resistant combined) *Escherichia coli* isolated from calves (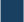; *n* = 195), cows (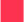; *n* = 202), and manure pits (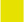; *n* = 196) from 101 dairy farms from Québec, Canada. Error bars represent 95% confidence intervals. Importance of antimicrobial for human medicine according to World Health Organization (15). *Statistically different (*p* < 0.05) probabilities of resistance between isolates obtained from calves, cows, or manure pit and estimated using either a logistic regression model with robust variance to account for clustering by farm (AMP, CHL, FIS, SPT, STR, SXT, TET), or a Fisher exact test (AMC, AZM, CRO, DAN, ENR, FOX, GEN, NAL, NEO, TIO). AMC, amoxicillin-clavulanic acid; AMP, ampicillin; AZM, azithromycin; CHL, chloramphenicol; CIP, ciprofloxacin; CRO, ceftriaxone; DAN, danofloxacin; ENR, enrofloxacin; FIS, sulfisoxazole; FOX, cefoxitin; GEN, gentamicin; MEM, meropenem; NAL, nalidixic acid; NEO, neomycin; SPT, spectinomycin; STR, streptomycin; SXT, trimethoprim-sulfamethoxazole; TET, tetracycline; TIO, ceftiofur.

The proportion of resistant isolates from calves exceeded 30% for several antimicrobials whereas it never reached 15% for any of the isolates from cows or manure pits (Figure 3). This difference between calves' isolates and those from other origin was statistically significant (*p* < 0.05) for 13 of the tested antimicrobials (amoxicillin/clavulanate, azithromycin, ampicillin, cefoxitin, ceftiofur ceftriaxone, chloramphenicol, neomycin, spectinomycin, streptomycin, sulfisoxazole, tetracycline and trimethoprim/sulfamethoxazole). There were no significant differences in proportion of resistant isolates between cow and manure pit isolates for all antimicrobials tested.

### Multidrug Resistance

**Figure 4:**
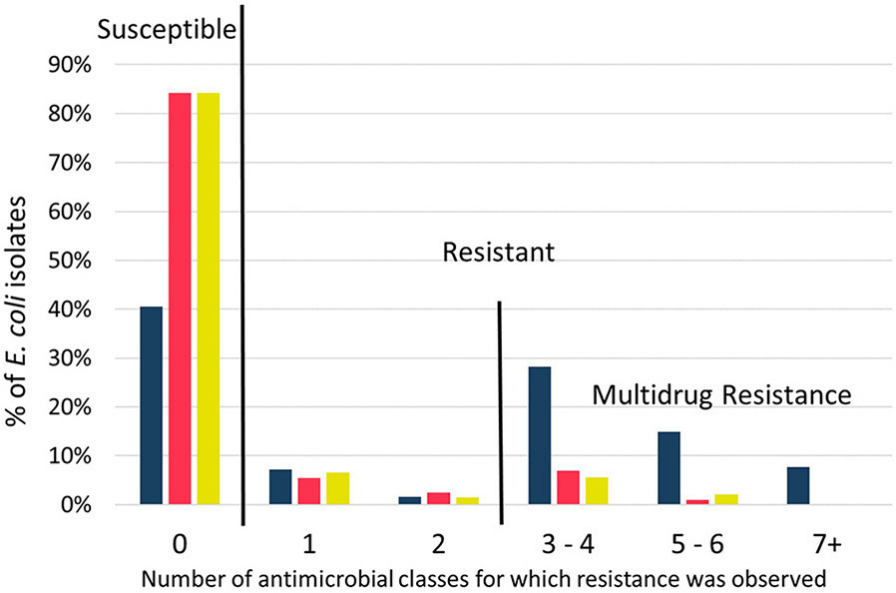
Distribution of susceptible and resistant profiles for *Escherichia coli* isolated from calves (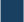; *n* = 195), cows (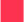; *n* = 202), and manure pits (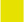; *n* = 196) from 101 dairy farms from Québec Province, Canada. Columns above zero were susceptible to all antimicrobials tested. Columns above a number > 0 were resistant to that corresponding number of antimicrobial classes. Multidrug resistance was defined by resistance to three or more antimicrobial classes. Globally, distribution of isolates from calves were statistically different from isolates of cows and manure pits (*p* < 0.05; Poisson regression with Tukey-kramer correction for multiple comparison).

The majority of isolates from cows (84%) and manure pits (84%) were susceptible to all antimicrobials tested (Figure 4). Prevalence of MDR *E. coli* was low for cows (8%) and manure pits (8%). However, approximately half (51%) of the *E. coli* isolates from calves were MDR with 8% being resistant to seven or more antimicrobial classes. One *E. coli* isolate from a calf was resistant to 9 classes of antimicrobials out of 11 tested and was considered extensively drug-resistant (19). It was only susceptible to quinolones and carbapenems. Sample types (calves, cows, manure pit) were significantly associated with number of antimicrobial classes for which resistance was observed (*p* < 0.01). Isolates from calves were resistant to 6.1 (95% CI 4.7–8.0) and 6.2 (95% CI 4.7–8.1) times more antimicrobial classes than isolates from cows and manure pits, respectively. Prevalence of MDR *E. coli* was high at the farm level (82%). There was no statistical difference in the number of resistances between isolates obtained during the autumn and spring sampling seasons.

### ESBL/AmpC Phenotypes and Genotypes

**Figure 5:**
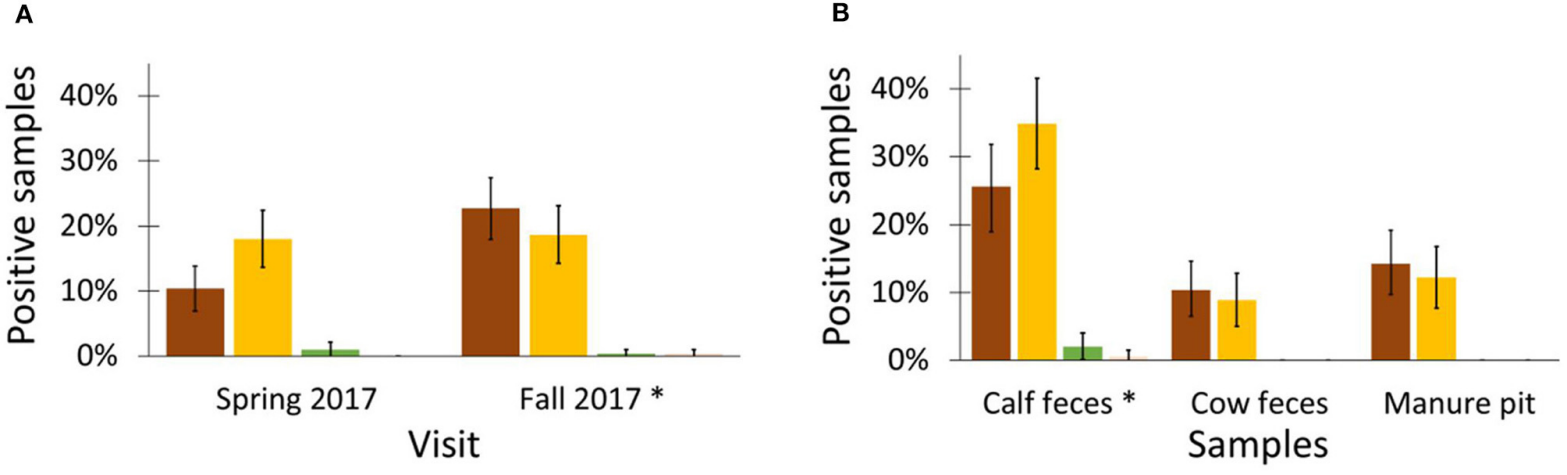
Distribution of phenotype ESBL (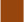), AmpC (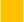), ESBL+AmpC (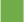), or other (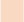) in *Escherichia coli* isolated from calves, cow and manure pits from 101 dairy farms from Québec Province, Canada**. (A)** Distribution of phenotypes by sampling seasons **(B)** Distribution of phenotypes by origin of sampling. *p < 0.05 Poisson distribution with Tukey-kramer correction for multiple comparison.

**Figure 6:**
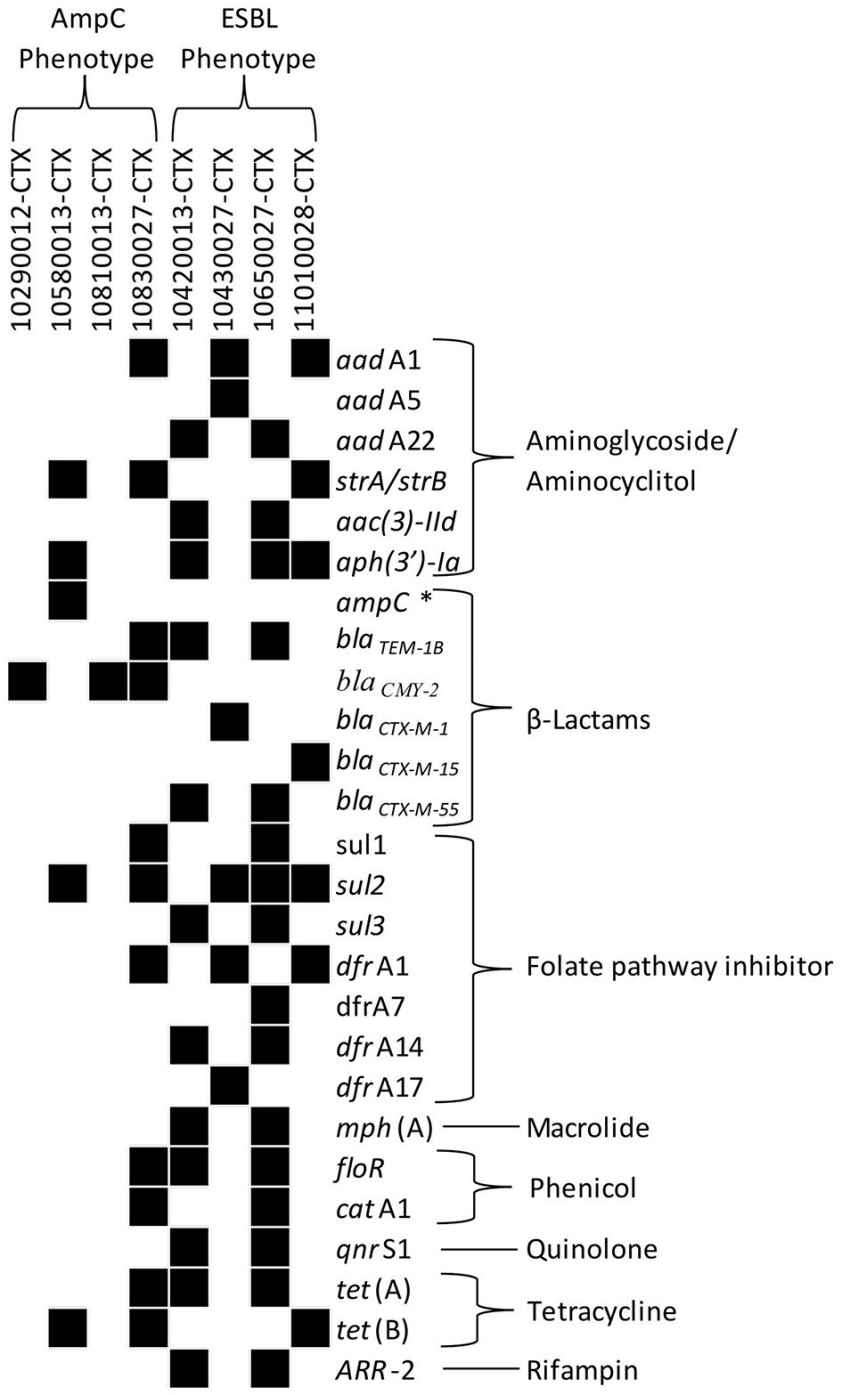
Antimicrobial resistance gene profiles of randomly selected ESBL (*n* = 4) or AmpC (*n* = 4) producing *Escherichia coli* isolated from calf or cow feces or manure pit of 101 dairy farms in Québec, Canada. Isolates were recovered using a selective protocol. Whole genome sequencing of selected isolates was processed on Resfinder and PointFinder in the Center for Genomic Epidemiology (CGE) to determine the presence of antimicrobial resistance genes. Black blocks indicate the presence of the corresponding gene. Antimicrobial classes associated with resistance genes are listed in the column to the right of the gene names. *Promoter mutation in *ampC*-42 C -> T.

Most ESBL/AmpC-producing *E. coli* were isolated from calf samples (Table 4). Overall, 85% (86/101) of farms were positive for ESBL/AmpC-producing *E. coli* in at least one sample during the study. The probability of retrieving a positive ESBL/AmpC phenotype within a sample was mainly influenced by the visit (47%), then the herd (33%), and, finally, by sample characteristics, such as its origin (20%), as determined by variance partitioning of the null model. Odds of retrieving an ESBL/AmpC-producing *E. coli* from calves were 16.9 (95% CI 7.5–38.1) and 10.1 (95% CI 4.8–21.2) times greater compared to cows and manure pits samples, respectively. Also, the recovery percentage of ESBL/AmpC-producing *E. coli* was statistically higher during the autumn season than in spring (1.9 times higher odds; 95% CI 1.3–2.8). There was no difference between the ESBL and AmpC phenotypes for each sampling season and origin of the sample (Figure 5). Commensal *E. coli* from samples that were positive for ESBL/AmpC had 3.0 (95% CI 2.5–3.5) times greater odds of carrying AMR than those that were negative for ESBL/AmpC. ESBL confirmation revealed four phenotypes: AmpC, ESBL, ESBL/AmpC, and other, in proportions of 51, 46, 2, and 1%; respectively. In total, 4 ESBL and 4 AmpC-producing *E. coli* isolates were sequenced using WGS to identify the genes responsible for this resistance. The *bla*_CTX−M_ gene was identified for the ESBL phenotype with the variants *bla*_CTX−M−1_ (*n* = 1), *bla*_CTX−M−15_ (*n* = 1), and *bla*_CTX−M−55_ (*n* = 2) (Figure 6). The resistance gene *bla*_CMY−2_ and a mutation in the promoter *ampC* (-42 C->T) known to cause increase in *ampC* expression (27) were both identified with regard to the AmpC phenotype (Figure 6).

**Table 4:** Number of positive samples with a presumptive ESBL/AmpC *Eschericha coli* isolated from calf or cow feces or manure pit of 101 dairy farms in Québec Canada.

**Sample types**	**Season**		**Total number (%)**
	**Spring (*n*= 299)**	**Autumn (*n* = 300)**	
	**Number**	**Number**	
Calves (*n* = 195)	53	70	123 (63)^a^
Cows (*n* = 202)	11	28	39 (19)^b^
Manure Pits (*n* = 202)	24	28	52 (26)^b^
Total Number (%)	88 (29)^a^	126 (42)^b^	214 (36)

*Values with different superscripts (a,b) within a row (for season) or within a column (for source) were statistically different using a generalized mixed model with Tukey-Kramer adjustment for multiple comparisons*.

## Discussion

The overall prevalence of *E. coli* AMR in this study was lower than in a report of the Canadian Integrated Program for Antimicrobial Resistance Surveillance (CIPARS) where 58% of isolates from beef cattle in slaughterhouses were susceptible to all antimicrobials tested (18). The most common resistances observed in CIPARS were toward tetracycline (36%), followed by sulfonamides (18%) and streptomycin (18%). In this latter report, prevalences were twice as high as those reported in our study for lactating cows and could mostly be explained by difference in source population (dairy cattle vs. beef cattle). Dairy farms located in Pennsylvania, USA, also observed a higher level of resistance toward tetracycline (93%), sulfonamide (56%) and streptomycin (53%) for fecal *E. coli* (28). However, it is difficult to compare prevalences because these authors used a preliminary screening step with antimicrobial-based selective medium before testing their *E. coli*. Interestingly, *E. coli* isolated from clinical mastitis in Canadian dairy farms (29) harbored similar AMR patterns and prevalence to those of cow fecal isolates in our study. The only difference was for the β-lactam antimicrobials that demonstrated higher levels of resistance in this latter study compared to those observed in our study. The majority of antimicrobial treatments (7 out of 8) approved for intramammary gland usage in Canada contain β-lactams and this likely explains the difference between results. Interestingly, we previously reported (14) that predominant antibiotic use was associated with penicillins, aminoglycosides, and polymyxins. This is partly related to the most observed resistance phenotypes in this study for streptomycin (an aminoglycosides). However, the highest level of resistance we observed toward tetracycline and sulfonamides were not associated with a high utilization of these antimicrobials. This could be partly explained because the antibiotics used most often on Quebec farms are intramammary (14). In addition, some mobile genetic elements present in *E. coli* were shown to allow the maintenance of resistance without the selection pressure by the antibiotic. This has been reported for sulfonamides genes, such as *sul*1 gene which is normally found linked to other resistance genes in class 1 integrons, whereas *sul*2 is usually located on small non-conjugative plasmids (30) or large transmissible multiresistance plasmids (31). Further analyses are required to demonstrate a clear correlation between antimicrobial usage and antimicrobial resistances. As for AMR genes, similar results were obtained by Tyson et al. (32) who reported a high prevalence for *str*A*/str*B, *sul*2 and *tet*(A) for cattle in the USA.

We found that the prevalence of AMR is highly dependent on origin of samples (cow, calf, or manure pit); the *E. coli* from dairy calves in our study carried proportionally higher levels of AMR. This is consistent with the current literature. Many reports have indicated an association between pre-weaned calves and increased risk of fecal shedding of resistant bacteria, compared to older animals on dairy farms (5, 8, 28, 35, 36). According to a review, the maximal prevalence of AMR carriage was around 2 weeks of age in calves not fed with any antimicrobial-containing milk replacer (5). Evidence, although reported as less consistent, of an impact of antimicrobial treatment via milk replacer feeding and feeding unsalable or waste milk on the presence of AMR bacteria was reported (5, 8, 37). However, fecal shedding of resistant bacteria did not increase when calves were fed colostrum from cows that received treatment at drying off (8, 34). The design of the current study could not investigate all the risk factors previously reported for pre-weaned calves. The exact cause of the increased shedding of resistant bacteria in calves is currently unknown. However, the commonly reported hypothesis is that calves have an underdeveloped gut in terms of bacterial diversity and resistant *E. coli* is able to compete successfully due to a possible linkage between resistance genes and genes conferring selective advantage in neonatal intestines. As the bacterial microbiota diversifies and increases in numbers, resistant *E. coli* loses its competitive advantage and is slowly removed from the gastrointestinal tract (38–41). In consequence, calves are colonized rapidly after birth by MDR *E. coli* (42), which is consistent with our results. Although calves pose a minimal risk to public health, they could be included in surveillance programs as a sentinel for AMR in dairy farms. However, the relatedness between resistant *E. coli* in calves, cows and the manure pit is still unclear.

Manure pits on dairy farms where animals are confined are used to store large amounts of raw feces mixed with used bedding materials and waste water. Raw feces originates mainly from cows and the contribution of calves is considered minor. This explains the alikeness between AMR results from cows and manure pits in our study. Even if there were fewer bacteria (difference in volume dilution for cows and manure pits), the proportions of AMR in *E. coli* from manure pits were not different from those found in adult cows. According to some studies, commercial spreading of dairy feces can disseminate AMR genes into the environment (33, 43, 44). Also, some authors reported that manure can be a reservoir of antimicrobial compounds, and via its application could increase the selection of resistant bacterial populations in soil (6). The possible dissemination to the environment and, subsequently, to other animals or humans of ESBL/AmpC producing *E. coli* present in the manure pit, as observed in the present study, is a public health concern. The main preoccupation is with ESBLs, which are frequently encoded by a resistance gene *bla*_*CTX*−*M*_ (45). These genes are associated with mobile genetic elements (46, 47) and it was reported that some *bla*_*CTX*−*M*_ recovered from *E. coli* isolated from food-producing animals were on plasmids and associated with other resistance genes (48). AMR environmental dissemination and persistence conveyed by livestock waste has recently been reviewed (49). Manure treatments such as thermophilic composting, biological treatments, and anaerobic digestion have been shown to reduce the number of antimicrobial-resistant bacteria (49).

Prevalence of ESBL/AmpC producing *E. coli* is variable between countries. A surveillance program from the European Union in 2015 reported that 43.6% (variation between 9 countries: 1.3–68.9%) of samples tested from calves under 1 year of age were carriers of ESBL/AmpC (20). In our study, the prevalence of ESBL/AmpC producing *E. coli* was 63% for calves, indicating a high level of colonization in young animals. This concurs with results from Belgium, France, Germany, Portugal, and Spain (20). In Canada, the frequency of fecal carriage of *E. coli* with reduced susceptibility to third-generation cephalosporins was 81.2% in Holstein dairy calves in New Brunswick, Canada (50). The difference in province of origin could explain the difference of prevalence noted in this study. The variation observed between different geographic locations could be due to a regional use of cephalosporins, which select for ESBL/AmpC producing *E. coli*. However, this correlation is not unanimous in the literature; some authors have reported an association between regular use of ceftiofur on the farm and fecal recovery of *E. coli* with reduced susceptibility to extended-spectrum cephalosporins (50). Furthermore, reduced use of 3rd and 4th generation cephalosporins was associated with a decrease in prevalence of ESBL/AmpC producing *E. coli* on Dutch dairy farms (41). On the other hand, some authors could not find a link between ceftiofur use on farms and probability of recovering ESBL/AmpC producing *E. coli* (51). In addition, use of ceftiofur for the treatment of respiratory diseases in dairy calves was statistically associated with decreased recovery of *E. coli* with reduced susceptibility to extended-spectrum cephalosporins (50).

We showed that dairy calves carried more ESBL/AmpC producing *E. coli* than their adult counterparts or the manure pit. This is consistent with a study in Holland where dairy calves demonstrated a much higher prevalence of ESBL/AmpC producing *E. coli* than cows (41). As explained above, dairy calves have an increased risk of fecal shedding of resistant bacteria including ESBL/AmpC producing *E. coli*.

Among the ESBL/AmpC producing *E. coli*, the predominant phenotype in Europe is ESBL for calves under 1 year of age (20). In North America, the predominant phenotype for dairies is unclear as there are few prevalence studies. However, some studies have reported that *bla*_CMY_ (associated with AmpC phenotypes) was predominant in dairy farms when compared to *bla*_CTX−M_ (associated with ESBL phenotypes) (28, 51). In our study, there was no difference between the prevalence of ESBL or AmpC phenotypes (51 and 46%, respectively). A specific geographic situation could explain why we found more ESBL producing *E. coli* compared to previous studies in North America in dairy cattle. However, a larger geographical study would be essential to confirm this difference. A temporal variation in the proportion of ESBL and AmpC could also explain the variation noted. Resistance genes associated with these phenotypes are dynamic over time. Over a decade ago, *bla*_*CMY*−2_ was prevalent and there were no reports of *bla*_*CTX*−*M*_ in animals in Canada (52). The first report of *bla*_*CTX*−*M*_ in animals in North America was in dairy cattle in Ohio in 2010 (53). Subsequently, numerous reports have indicated the presence of *bla*_*CTX*−*M*_ in animals. There is a public health concern for transmission of these resistance genes associated with MDR plasmids between bacteria from humans, animals, and the environment.

We found that ESBL/AmpC producing *E. coli* were recovered more frequently in the autumn season. This difference is not caused by outdoor temperature because there is no difference for the manure pit samples between the two seasons. Seasonal variation in excretion of ESBL/AmpC is poorly described in veterinary medicine. A study in a human population reported that carriage of ESBL *E. coli* and *Klebsiella pneumoniae* was highest in the months at the end of summer/beginning of autumn (August/September) (54). Another study reported higher levels of excretion of ESBL between July and September, although this was inversely correlated with use of antimicrobials (55). However, some factors that could explain this variation in prevalence, such as outdoor activity, different feed preparation methods, and increased movement, are not applicable for dairies in Québec (56). Nevertheless, even if animals did not have access to the outside for the majority of farms, buildings were often opened to allow better aeration during the summer. Consequently, many animal species such as birds had access to the interior of the buildings. It has been shown that migratory birds may carry ESBL *E. coli* (57). We could hypothesize that the opening of buildings during the summer allows wild animals potentially carrying ESBL/AmpC-producing *E. coli* to be in contact directly or indirectly with farm animals. In the context of our results and of manure application in Canada, it is tempting to speculate that spring manure application might be a better strategy in terms of reducing AMR spread associated with ESBL/AmpC resistance.

We observed a statistical association between presence of ESBL/AmpC-producing *E. coli* as recovered by the selective protocol and MDR in commensal *E. coli* from the same sample. This observation could be associated with farm characteristics. Risk factors such as use of antimicrobials are farm dependant and could lead to a selection of resistant bacteria in the enteric gut of animals. This selection leads to a larger number of resistant bacteria, but also the presence of ESBL/AmpC-producing *E. coli*. This observation warrants further investigation.

In this cross-sectional study, the random selection of herds allowed an accurate estimate of the target population (the province of Québec). However, studies that require enrollment of participants are inherently prone to selection bias (58). Indeed, it is possible that producers who have experienced medical problems in their herd requiring antimicrobial treatment may be more prone to decline participation in this project because they would be uncomfortable to be identified as heavy users of antimicrobials. Nevertheless, Lardé et al. (14) demonstrated that the herds that accepted or refused to participate in the current study were not different in terms of herd size, amount of owned milk quota, and average daily production.

In conclusion, our results suggest that AMR in dairy farms from Québec province, Canada, is low for highest priority critically important antimicrobials for humans. As previously described, pre-weaned calves carried higher levels of AMR in commensal fecal *E. coli* than cows and manure pit systems on dairy farms. Also, there is a high prevalence of ESBL/AmpC-producing *E. coli* on farms, particularly in calves. Presence of ESBL *E. coli* in manure pits, although low, nevertheless suggests a possible contamination of the environment and raises public health concern.

## Data Availability Statement

The whole genome sequences presented in this study can be found in online repositories. The names of the repository and accession number can be found at: https://www.ncbi.nlm.nih.gov/bioproject/PRJNA716674/.

## Ethics Statement

The animal study was reviewed and approved by Animal Use Ethics Committee of the Université de Montréal (Protocol 16-Rech-1859). Written informed consent was obtained from the owners for the participation of their animals in this study.

## Author Contributions

MA, DF, SD, and J-PR conceived research projet and designed experiments. JM and HL recruited dairy farms and coordinated sample collection. JM designed protocols and conducted experiments. JM, MA, SD, and JF contributed to data analysis and interpretation. JM wrote the draft manuscript and MA, SD, JF, J-PR, HL, and SD critically revised it. All authors contributed to the article and approved the submitted version.

## Conflict of Interest

The authors declare that the research was conducted in the absence of any commercial or financial relationships that could be construed as a potential conflict of interest.
